# Corn distiller's dried grains with solubles as an alternative ingredient to corn and soybean meal in Pekin duck diets based on its predicted AME and the evaluated standardized ileal digestibility of amino acids

**DOI:** 10.1016/j.psj.2022.101974

**Published:** 2022-05-21

**Authors:** X.M. Ding, Y.Y. Qi, K.Y. Zhang, G. Tian, S.P. Bai, J.P. Wang, H.W. Peng, L. Lv, Y. Xuan, Q.F. Zeng

**Affiliations:** ⁎ Institute of Animal Nutrition, Sichuan Agricultural University, Chengdu, Sichuan, China; † Key Laboratory for Animal Disease-Resistance Nutrition of, Ministry of Education, Ministry of Agriculture and Rural Affaires, Sichuan Province 611130, China

**Keywords:** DDGS, ducks, meat quality, serum biochemical parameters, standard ileal amino acid digestibility

## Abstract

The study aimed to investigate the effects of dietary distillers dried grains with solubles **(DDGS**) levels on growth performance, carcass characteristic, serum biochemical indexes, meat physical and chemical quality, nutrient utilization, and standardized ileal digestibility of amino acids **(SIDAA**) in Pekin ducks aged 11 to 42 d based on the evaluation of its SIDAA. A total of 560 eleven-day-old Cherry Valley ducks were randomly allotted to 5 treatments with 7 replicate pens per treatment and 16 ducks per pen based on the average body weight. Six isonitrogenous and isocaloric experimental diets were formulated on a digestible amino acid basis to produce diets containing 0, 5, 10, 15, and 20% DDGS, respectively. With increasing of dietary DDGS levels, a linear and quadratic reduction (*P* < 0.05) was observed in the body weight (**BW**) at d 42, average day gain (**ADG**) and average day feed intake (**ADFI**) from d 11 to 42, breast meat yield, the moisture and protein content in the breast meat, and dietary DM and EE utilization. Moreover, a linear and quadratic increase (*P* < 0.05) was observed in the b* value of the breast meat and serum total cholesterol and triglyceride concentrations. Compared with the control group, the group with 10% inclusion of DDGS exhibited no adverse effect on growth performance, carcass characteristics, serum biochemical indexes, meat physical and chemical quality, nutrient utilization, and the SIDAA of the diets (*P* > 0.05). These results suggested that 10% of corn DDGS can function as an alternative ingredient to corn and soybean meal, and the optimal levels of DDGS in the diets of ducks aged from 11 to 42 d depends more on its quality.

## INTRODUCTION

In recent years, the sharp increase in the price of corn and soybean accompanied by their limited supply has become grave concern in the poultry feed industry. Moreover, corn is used as the energy source in poultry diets, leading to more and fiercer competition of the feed-food competition due to the increasing demand for bio-based ethanol (Renewable Fuel Association [**RFA**], 2019). Distiller's dried grain with solubles (**DDGS**) is a byproduct of dry-grind ethanol production (Cromwell et al., 1993), which is rich in crude protein (**CP**), fat, fiber, vitamins, and minerals and is currently used as feed stuffs for aquaculture, livestock, as well as poultry (Iram et al., 2020). An urgent need to evaluate and scientifically utilize some unconventional feedstuffs, such as DDGS, was initiated to cope with the feed-food competition.

Many previous studies have suggested that the maximum amount of DDGS was 15 to 24% in the broiler chickens’ diet (Lumpkins et al.,2004; Wang et al., 2007; Min et al., 2012) and 15 to 25% in the Pekin ducks’ diet (Kowalczyk et al.,2012; Xie et al.,2016; Zhai et al., 2020). However, due to variations in the growing conditions, ethanol processing methods, and extraction of oil, the nutrient composition of DDGS from different sources varies widely (Meloche et al. 2013, 2014). As a result, inclusion of DDGS-dose in poultry feeds has been controversial and varied with the age of the birds and the breeders (Iram et al., 2020). How to precisely use DDGS in poultry diets still needs more concerns.

Evaluating the apparent metabolizable energy (**AME**) and the standardized ileal digestibility of amino acids (**SIDAA**) of unconventional feedstuffs is critical for utilizing these feeds in poultry diets. The AME concentration, SIDAA content, and diet pattern hugely effect poultry feed intake and growth rate (Zeng et al., 2015a; Adeola, 2006). However, limited information is available about the dosage of DDGS inclusion in Pekin duck diets based on the evaluation of AME and SIDAA content of DDGS. In a dose-response study, one or more parameters (e.g., growth performance, serum biochemical indices, meat yield, and quality) are estimated for particular poultry. The maximum safe level of the test ingredient is determined for each parameter (Alhotan and Pesti, 2016). Li et al. (2012) reported that feeding geese diets containing corn DDGS accelerates the lipid oxidation in muscle, causing rancidity. This happens because corn DDGS has high unsaturated fatty acids, mainly oleic acid, and linoleic acid, which are prone to oxidation (Hanson et al., 2015). Therefore, an experiment with a dose-response design was conducted to evaluate DDGS as an alternative ingredient to corn and soybean meal in Pekin duck diets. The effect of DDGS was assessed based on its AME and SIDAA content on the growth performance, meat quality, serum indices, and nutrient utilization of the ducks.

## MATERIALS AND METHODS

The Institutional Animal Care and Use Committee of Sichuan Agricultural University approved all procedures used in the study.

### Evaluation of Standardized Ileal Digestibility of Amino Acids of DDGS

The substitution method to evaluate the SIDAA of DDGS in Pekin ducks was performed according to Zhang et al. (2020). Briefly, a total of 42 twenty-two-day-old male ducks were weighed and allocated to 3 dietary treatment groups with seven replicate cages (2 ducks/cage) based on the similar average body weight. The 3 diets included a basal diet, a DDGS diet (15% DDGS: 85% basal diet), and a nitrogen-free diet. All diets contained 0.5% titanium dioxide (**TiO_2_**) as an indigestible marker. The ducks were acclimated for 3 d, and then experimented with for 4 d. On d 29, the ducks were euthanized by cervical dislocation to collect the ileal digesta. The analyzed nutrient composition, predicted AME, and evaluated SIDAA of DDGS are presented in Table 1.

**Table 1:** Analyzed nutrient composition, AME, and SIDAA of corn distillers dried drains with solubles.

Items		Analyzed composition	Evaluated value
Gross energy, MJ/kg		19.41	-
AME, MJ/kg		-	11.16^1^
Dry matter, %		80.75	
Crude protein, %		17.48	
Crude fat, %		11.26	
Aflatoxin B1		Not detected	
Zearalenone, μg/kg		20.60	
Deoxynivalenol, μg/kg		400.00	
Nonessential amino acids, g/kg			SIDAA, %
Aspartic acid		1.59	77.58
Alanine		1.66	82.59
Cysteine		0.26	79.71
Glutamic acid		4.60	84.47
Glycine		0.87	72.94
Proline		2.18	85.31
Serine		1.39	82.76
Tyrosine		0.92	80.59
Essential amino acid, g/kg			
Arginine		0.91	80.41
Histidine		0.64	83.75
Isoleucine		0.87	78.38
Leucine		2.89	84.73
Lysine		0.68	75.06
Methionine		0.41	86.51
Phenylalanine		1.06	83.14
Threonine		1.02	79.26
Valine		1.09	79.01

Abbreviations: AME, apparent metabolizable energy; SIDAA, standardized ileal digestibility of amino acids.

1 The AME of DDGS was calculated based on the prediction equation (AME (MJ/kg) = 0.230EE + 8.573), which reported in our previous study of Shu et al. (2019).

### Birds, Diets, and Management

A total of 600 one-day-old Cherry Valley ducks were fed a standard starter diet containing 12.13 MJ/ kg ME, 19.5% CP, 1.15% lysine, 0.48% methionine, 0.78% threonine, and 0.22% tryptophan from d 1 to d 10 after hatching. The experiment was performed from d 11 to 42. On d 11, 560 ducks were randomly assigned to 5 treatment groups with 7 replicate pens per treatment and 16 ducks per pen based on the average BW. Ducks were reared in the pens in a temperature and humidity-controlled room, and had free access to water and feed up to d 42. Five isonitrogenous and isocaloric experimental diets were formulated based on the DDGS's AME and SIDAA (Table 1) to produce diets containing 0, 5, 10, 15, and 20% DDGS, respectively. Diets were fortified with synthetic feed-grade lysine, methionine, threonine, and tryptophan to provide the recommended levels of AA for Pekin ducks in accordance with NRC (1994) and Zeng et al. (2015b) (Table 2).

**Table 2:** Composition and nutrient contents of the experimental diets (air dry basis) %.

Items	DDGS levels, %				
	0	5	10	15	20
Ingredients, %					
Corn	64.77	65.86	62.64	59.82	58.23
Soybean oil	0.97	0.65	0.85	1.05	1.20
Soybean meal	18.31	16.13	13.10	10.22	7.60
Rapeseed meal	6.00	6.00	6.00	6.00	6.00
Wheat middling	6.10	2.50	3.50	4.00	3.00
DDGS	0.00	5.00	10.00	15.00	20.00
L-Lysine. HCL	0.13	0.17	0.22	0.27	0.32
Threonine	0.05	0.07	0.05	0.06	0.06
Tryptophan	0.04	0.02	0.06	0.06	0.07
DL-Methionine	0.19	0.18	0.18	0.16	0.16
Calcium carbonate	0.87	0.93	1.00	1.08	1.14
Dicalcium phosphate	1.55	1.47	1.38	1.25	1.19
Sodium chloride	0.35	0.35	0.35	0.35	0.35
Choline chloride (50%)	0.15	0.15	0.15	0.15	0.15
Vitamin premix^1^	0.03	0.03	0.03	0.03	0.03
Mineral premix^2^	0.50	0.50	0.50	0.50	0.50
Total	100	100	100	100	100
Price, RMB/kg	2.67	2.66	2.67	2.67	2.68
Calculated nutrients levels, %					
AME, MJ/kg	12.15	12.15	12.15	12.15	12.15
Crude protein	16.5	16.5	16.5	16.5	16.5
Crude fiber	3.30	3.28	3.45	3.60	3.68
Calcium	0.76	0.76	0.76	0.76	0.76
Available phosphorus	0.38	0.38	0.38	0.38	0.38
Lysine	0.90	0.90	0.90	0.90	0.90
Methionine	0.43	0.43	0.43	0.43	0.43
Threonine	0.69	0.69	0.69	0.69	0.69
Tryptophan	0.19	0.19	0.19	0.19	0.19

1 Vitamin premix provides the following per kg of final diet: vitamin A 8,000 IU; vitamin D_3_ 2,000 IU; vitamin E 5 mg; vitamin K_2_ 1 mg; vitamin B_1_ 0.6 mg; vitamin B_2_ 4.8 mg; vitamin B_6_ 1.8 mg; vitamin B_12_ 0.009 mg; niacin 10.5 mg; DL-calcium pantothenate 7.5 mg; folic acid 0.15 mg.

2 Mineral premix provides the following per kg of final diet: Fe (FeSO_4_·H_2_O) 80 mg; Cu (CuSO_4_·5H_2_O) 8 mg; Mn (MnSO_4_·H_2_O) 70 mg; Zn (ZnSO_4_·H_2_O) 90 mg; I (KI) 0.4 mg; Se (Na_2_SeO_3_) 0.3 mg.

### Data Collection and Measurements

On d 42, the body weight (**BW**) and feed consumption of ducks were recorded for each pen. Feed intake (**FI**), BW gain (**BWG**), and feed-to-gain (**F: G**) ratio were determined. Birds that died during the experiment were weighed, and the data were used to calculate the F:G ratio.

Further, two birds with BW closer to the mean were selected from each pen, and 5 mL blood was collected from jugular vein. The whole blood samples were centrifuged at 3,000 *g* for 15 min at 4°C to collect the serum, and then stored at –20°C until biochemical parameters were analyzed. Serum aspartate aminotransferase (**AST**), alanine aminotransferase (**ALT**) activities, glucose, high density lipoprotein cholesterol (**HDL-c**), low density lipoprotein cholesterol (**LDL-c**), total cholesterol (**TC**), triglyceride (**TG**), total protein (**TP**), and uric acid (**UA**) were analyzed using an automatic biochemical analyzer (HITACHI 7180, Japan).

After that, the ducks were euthanized by carbon dioxide asphyxiation and the carcasses were harvested. The feathers were plucked, and evisceration was performed to obtain dressed breast and leg meats. Carcass yield was determined as the carcass weight in relation to BW expressed as a percentage of BW (%), whereas breast and leg meat weights were expressed as percentages of the carcass weight. Meanwhile, breast meat from both sides of the carcass were skinned and deboned to estimate carcass traits. Breast meat physical parameters included meat color (L*, a*, b*), pH value, tenderness and drip loss, and meat chemical parameters include moisture, CP, and fat content were determined according to the method of Qi et al. (2022).

### Assay of Nutrient Utilization and SIDAA of diets

On d 22, two birds per pen were randomly selected (12 ducks per treatment, 72 ducks in total) and transferred to metabolic cages (2 ducks per cage) and fed with the original diets mixed with titanium dioxide (**TiO2**; 0.5%). After acclimation for 3 d, on d 25 at 08:00 h, the excreta were collected for 3 successive days (72 h; collected every two hours and pooled by a cage) to determine the excreta dry matter (**DM**), energy, CP, and ether extract (**EE**) retention. On d 28 at 08:00 h, the birds were euthanized using CO_2_ and the digesta from the terminal two-thirds of the ileum were collected to determine the SIDAA of the experimental diets. The ileum was defined as that portion of the small intestine extending from Meckel's diverticulum to a point 40 mm proximal to the ileocecal junction.

### Statistical Analysis

Data were analyzed using One-way ANOVA of SPSS Statistics V22.0 (SPSS software for Windows, release 22.0, SPSS Inc., Chicago, IL). Duncan's test was used for multiple comparisons, and orthogonal polynomials were used to test linear and quadratic changes with increasing DDGS levels in the diet. Significance was declared at *P <* 0.05*.*

## RESULTS

### Growth Performance

The effects of dietary supplementation by DDGS on BW, BWG, FI, and F:G ratio is presented in Table 3. Dietary DDGS levels had no significant effect (*P* > 0.05) on the ADFI, F:G ratio, and mortality of the meat ducks during the experimental period. However, the BW (d 42), ADG (d 10–42), and ADFI (d 10–42) presented a linear and quadratic decrease (*P* < 0.05) with the increase of dietary DDGS levels. In addition, compared with other groups, ducks fed 20% DDGS had a significantly decreased (*P* < 0.05) in the BW (d 42) and ADG (d 10–42). Compared with the control group, 5% DDGS group numerically increased (*P* > 0.05) the BW (d 42) and ADG (d 10–42).

**Table 3:** Effects of dietary DDGS levels on growth performance of ducks from 11 to 42 d of age.

Item^1^	Dietary DDGS levels, %					SEM	*P*-value		
	0	5	10	15	20		ANOVA	Linear	Quadratic
10 d BW, g	403.1	401.0	400.4	402.2	396.0	4.25	0.508	0.255	0.389
42 d BW, g	3,336^ab^	3,356^a^	3,283^ab^	3,215^bc^	3,132^c^	63.40	0.008	0.001	0.002
10–42 d ADG, g	91.67^ab^	92.36^a^	90.09^ab^	87.91^bc^	85.49^c^	1.97	0.009	0.001	0.002
10–42 d ADFI, g	201.0	202.9	200.8	198.3	191.8	4.51	0.146	0.027	0.028
F: G, g/g	2.20	2.20	2.23	2.26	2.24	0.04	0.509	0.159	0.356
Mortality, %	4.46	3.57	1.79	8.93	6.25	3.77	0.403	0.295	0.577

Abbreviations: BW, body weight; ADG, average day gain; ADFI, average day feed intake.

1 Values are the means of 6 replicates of 16 ducks each.

a-c Values within a row with no common superscripts differ significantly (*P* < 0.05).

### Carcass Traits

A graded increase in the DDGS levels in the diets resulted in a linear and quadratic decrease (*P* < 0.05; Table 4), in the weight of carcass, eviscerated with giblet, eviscerated, breast meat, and breast meat percentage. And compared with other groups, the 20% DDGS group significantly decreased (*P* < 0.05) the weight of eviscerated with giblet, eviscerated, and breast meat. There were no linear or quadratic effects (*P* > 0.05) on the leg meat and abdominal fat.

**Table 4:** Effects of dietary DDGS levels on carcass traits of ducks at 42 d of age.

Item^1^	Dietary DDGS levels, %					SEM	*P*-value		
	0	5	10	15	20		AVONA	Linear	Quadratic
Absolute weight of carcass traits, g									
Carcass	2,822	2,869	2,810	2,829	2,666	73.6	0.090	0.029	0.016
Eviscerated with giblet	2,688^a^	2,696^a^	2638^ab^	2,675^a^	2,501^b^	67.6	0.041	0.027	0.009
Eviscerated	2,429^a^	2,442^a^	2,399^a^	2,410^a^	2,260^b^	63.7	0.050	0.031	0.005
Breast muscle	346.0^a^	351.0^a^	330.7^a^	315.9^a^	271.4^b^	21.1	0.005	0.001	0.001
Leg muscle	278.0	264.7	262.6	262.0	249.6	14.2	0.414	0.144	0.328
Abdominal fat	30.29	35.43	34.14	35.00	32.71	4.68	0.810	0.753	0.528
Relative weight of carcass traits, %									
Carcass	84.42	85.72	86.45	86.24	85.82	1.12	0.426	0.556	0.585
Eviscerated with giblet	80.39	80.56	81.15	81.55	80.52	0.87	0.627	0.445	0.282
Eviscerated	72.66	72.97	73.82	73.47	72.75	0.89	0.654	0.467	0.106
Breast muscle	10.34^a^	10.48^a^	10.15^a^	9.61^ab^	8.72^b^	0.51	0.011	0.003	0.002
Leg muscle	8.31	7.91	8.10	8.00	8.04	0.44	0.917	0.881	0.936
Abdominal fat	0.90	1.06	1.05	1.06	1.05	0.13	0.709	0.389	0.473

1 Values are the means of 6 replicates of 16 ducks each.

a-b Values within a row with no common superscripts differ significantly (*P* < 0.05).

### Meat Physical and Chemical Quality

The effects of dietary DDGS levels on the meat's physical and chemical quality are displayed in Table 5. There were no significant effects (*P* > 0.05) on the drip loss, shear force, pH, color, and fat content in the breast meat among all treatments. However, a linear and quadratic decrease (*P* < 0.05) was observed in the CP content in breast meat upon increasing the dietary DDGS levels. Also, a linear and quadratic increase (*P* < 0.05) was observed in moisture content in the breast meat and b* with increasing dietary DDGS levels.

**Table 5:** Effects of dietary DDGS levels on meat quality of ducks at 42 d of age^1^.

Item^1^	Dietary DDGS levels, %					SEM	*P*-value		
	0	5	10	15	20		ANOVA	Linear	Quadratic
Breast meat physical quality									
Drip loss, %	7.54	6.87	3.54	5.98	7.13	2.48	0.853	0.680	0.663
Shear force, (kgf/cm^2^)	4.22	4.50	3.75	3.30	4.49	0.75	0.450	0.608	0.447
45 min pH value	5.90	5.89	5.91	5.99	6.04	0.11	0.600	0.229	0.203
24 h pH value	5.81	5.84	6.04	6.03	6.03	0.15	0.386	0.095	0.235
45 min color									
L*	52.01	55.32	53.64	50.09	55.13	1.94	0.058	0.673	0.905
a*	14.14	12.04	12.91	12.30	10.39	1.65	0.271	0.049	0.145
b*	6.15	7.43	6.73	7.31	9.21	1.01	0.057	0.030	0.046
24 h color									
L*	54.66	54.28	51.23	51.77	55.28	1.87	0.138	0.875	0.181
a*	12.59	11.94	13.16	11.73	10.65	0.95	0.121	0.109	0.120
b*	5.02	5.72	5.45	5.32	7.36	0.84	0.071	0.048	0.068
Breast meat chemical quality									
Moisture, %	77.42	78.05	78.12	78.48	78.51	0.50	0.221	0.020	0.041
Protein, %	20.17	19.99	19.72	19.64	18.79	0.50	0.071	0.007	0.017
Fat, %	6.11	6.18	5.42	5.96	6.60	0.54	0.324	0.348	0.165

L*: Lightness; a*: Redness; b*: Yellowness.

1 Values are the means of 6 replicates of 16 duck each.

### Serum Biochemical Indices

No significant effects (*P* > 0.05) were observed on the contents of ALT, AST, glucose, HDL-c, LDL-c, TP, and UA in the serum among all groups (Table 6). However, the TC content in the 20% DDGS groups was significantly higher (*P* < 0.05) than that in the 0 and 5% DDGS groups. Moreover, the serum TG contents in the 0 and 5% DDGS groups were significantly lower (*P* < 0.05) than in the 15 and 20% DDGS groups.

**Table 6:** Effects of dietary DDGS levels on serum biochemical indices of ducks at 42 d of age.

Item^1^	Dietary DDGS levels, %					SEM	*P*-value		
	0	5	10	15	20		ANOVA	Linear	Quadratic
ALT, U/L	27.44	23.20	22.80	26.30	23.48	3.15	0.490	0.564	0.809
AST, U/L	21.12	17.94	30.39	21.11	23.92	6.03	0.327	0.579	0.649
Glucose, mmol/L	7.03	7.69	7.03	6.75	6.56	0.66	0.514	0.063	0.147
HDL-C, mmol/L	3.30	3.38	3.04	3.09	3.33	0.29	0.717	0.697	0.901
LDL-C, mmol/L	0.55	0.72	0.77	0.66	0.89	0.11	0.051	0.034	0.108
TC, mmol/L	5.51^b^	5.67^b^	5.99^ab^	5.93^ab^	6.25^a^	0.25	0.050	0.007	0.025
TG, mmol/L	0.77^b^	0.74^b^	1.14^ab^	1.32^a^	1.28^a^	0.21	0.020	0.005	0.021
TP, g/L	29.99	30.43	32.31	30.90	30.20	1.33	0.438	0.941	0.310
UREA, mmol/L	0.49	0.53	0.59	0.49	0.56	0.08	0.691	0.379	0.435

Abbreviations: ALT, alanine aminotransferase; AST, aspartate aminotransferase; HDL-C, high density lipoprotein cholesterol; LDL-C, low density lipoprotein cholesterol; TC, total cholesterol; TG, triglyceride; TP, total protein; UREA, uric acid.

1 Values are the means of 6 replicates of 16 duck each.

a–b Values within a row with no common superscripts differ significantly (*P*< 0.05).

### Dietary Nutrient Utilization and SIDAA

As shown in Table 7, the utilization of dietary DM and EE linearly or quadratically decreased with an increase in the dietary DDGS levels (*P* < 0.05). However, the energy availability in the 15% DDGS group was significantly lower than in other groups. There was no significant different (*P* > 0.05) in the effect of dietary DDGS levels on the CP availability and SIDAA among all groups.

**Table 7:** Effects of dietary DDGS levels on nutrient utilization and standardized ileal digestibility of amino acids in Pekin ducks.

Item^1^	Dietary DDGS levels, %					SEM	*P*-value		
	0	5	10	15	20		ANOVA	Linear	Quadratic
Nutrient utilization, %									
Dry matter	79.53^a^	78.92^ab^	79.39^ab^	75.81^c^	77.03^bc^	1.09	0.01	0.00	0.02
Energy	82.28^a^	81.71^a^	82.11^a^	79.66^b^	81.48^a^	0.79	0.02	0.07	0.13
CP	79.28	77.86	77.57	75.99	76.3	1.98	0.49	0.08	0.19
EE	94.43^a^	94.05^a^	91.20^b^	93.81^a^	91.69^b^	0.77	0.00	0.01	0.03
Essential amino acid (EAA),%									
Arg	83.05	78.36	79.57	80.24	79.48	4.39	0.863	0.589	0.699
His	85.21	81.05	81.69	82.73	81.90	3.67	0.816	0.546	0.629
Ile	79.93	75.85	76.11	77.42	78.17	5.48	0.946	0.832	0.741
Lys	78.05	70.92	73.15	75.49	75.59	5.79	0.783	0.938	0.626
Leu	84.55	81.70	83.11	84.97	86.12	3.85	0.837	0.511	0.568
Met	84.36	83.23	81.40	87.27	85.74	4.44	0.733	0.516	0.668
Phe	83.39	80.34	81.12	82.96	83.53	4.23	0.925	0.803	0.729
Val	80.05	75.91	77.01	78.27	78.76	5.17	0.943	0.949	0.781
Thr	80.15	75.76	75.56	78.74	77.22	5.42	0.900	0.806	0.777
Total EAA	82.01	77.93	78.87	80.89	81.04	4.64	0.902	0.960	0.729
Non-essential amino acids (NEAA), %									
Ala	82.43	79.38	80.94	82.75	83.24	4.26	0.904	0.640	0.718
Asp	81.76	76.83	77.51	76.60	77.37	4.41	0.764	0.343	0.455
Cys	80.53	76.87	77.07	79.26	80.37	4.18	0.853	0.894	0.574
Gly	75.04	69.38	70.58	72.02	71.55	5.24	0.858	0.693	0.681
Glu	85.83	82.67	82.85	83.47	84.24	3.15	0.856	0.682	0.563
Pro	86.19	82.76	82.41	84.14	83.38	3.07	0.754	0.529	0.532
Tyr	80.99	76.48	77.97	80.18	81.29	5.57	0.900	0.783	0.687
Ser	83.62	79.52	80.90	82.07	82.05	4.55	0.923	0.927	0.791
Total NEAA	80.10	75.61	76.25	76.99	77.30	4.44	0.875	0.640	0.635
Total AA	80.98	76.68	77.47	78.82	79.06	4.52	0.899	0.830	0.700

Abbreviations: Arg, arginine; Ala, alanine; Asp, aspartic acid; Cys, cysteine; CP, crude protein; EE, ether extract; Gly, glycine; Glu, glutamic acid; His, histidine; Ile, isoleucine; Lys, lysine; Leu, Leucine; Met, Methionine; Phe, phenylalanine; Pro, proline; Ser, serine; Thr, threonine; Tyr, tyrosine; Val, valine.

1 Values are the means of 6 replicates of 2 ducks each per treatment.

a–c Values within a row with no common superscripts differ significantly (*P*< 0.05).

## DISCUSSION

The AME of the experimental DDGS was 11.16 MJ/kg via calculation based on the prediction equation (AME [MJ/kg] = 0.230 EE +8.573) in the Pekin ducks from our previous study by Shu et al. (2020). This value was in line with the average nitrogen-corrected AME (**AMEn**) value of 11.2 MJ/kg (range from 9.0 to 13.0 MJ/kg) obtained by Rochell et al. (2011) from 6 sources of DDGS in broiler chicks. Meloche et al. (2014) also reported an average AME value of 11.6 MJ/kg (range of 8.3–15.2 MJ/kg) from 15 sources of DDGS in broiler chicks. These supporting reports indicated the reliability of the predicted AME of DDGS in the present study.

Notably, the SIAAD of essential AAs was greater in the current study than the reported values for DDGS in broiler chickens (Adedokun et al. 2015). For example, Lys: 75.06 vs. 58.32%, Met: 86.51 vs. 81.66%, Arg: 80.41 vs. 79.18%, Thr: 79.26 vs. 65.68%, and Val: 79.01 vs. 72.62%. In the study on broiler chickens by Batal and Dale (2006), the values for Lys (75.06 vs. 69.60%) and Thr (79.26 vs. 74.5%) were higher, while the values for Met (86.51 vs. 86.80%) and Val (79.01 vs. 79.50%) were similar. This discrepancy between results could be due to differences in the breeding conditions of the birds and the evaluation methods. Overall, the DDGS analyzed in this study had better, or similar SIDAA or digestibilities than varying sources of DDGS reported elsewhere. More importantly, the AAs in DDGS are approximately 3-fold concentrated compared to whole corn grain. The average AA profile of corn contains 0.2% Lys and 0.2% Met, while DDGS contains 0.7% Lys and 0.5% Met. The DDGS used in this study contained 0.68% Lys and 0.41% Met. Although corn had a higher SIDAA of Lys (92.4%) and Met (95.0%) than that of DDGS (Adedokun et al., 2015), DDGS was a better source of SIDAA for ducks.

The results of the feeding experiment indicated that DDGS could be included in the diet at 10% dosage without causing any negative impact on the growth performance, carcass traits, breast meat yield, physical and chemical quality, serum parameters, and nutrient utilization (except for EE availability), and SIDAA of the ducks. These results were in disagreement with the reported value of 20% inclusion of liquor distiller's grains with solubles in the study by Zhai et al. (2020) on ducks, 15% sorghum DDGS in the study by Xie et al. (2016) on Micro duck drakes, and 25% maize DDGS in the study by Kowalczyk et al. (2012) on ducks. Loar et al. (2012) evaluated the effects of feeding 0 vs. 8% DDGS during the starter and grower phases (d 0–14 and d 14–28) and subsequently feeding a finisher diet (d 28–42) with 0, 7, 14, 21, or 28% DDGS on broilers. They found a linear decrease in the dressing percentage and breast meat yield upon increasing the inclusion levels of DDGS. This disagreement could be due to the different sources of DDGS, such as corn, wheat, and barley, or the quality of DDGS.

Indeed, in the present study, the inclusion rates of 15 and 20% DDGS presented negative effects on the growth rate, breast meat yield, the moisture and protein content in breast meat, serum TG and TC concentrations, and the availability of dietary DM and EE. One reason might be the approximately 3 times higher fiber content of DDGS than that found in corn and soybean meal (Spiehs et al., 2002), which may result in reduced growth performance and dressing percentage (Pond et al., 1989). Moreover, Rodrigues and Chin (2012) suggested that the presence of toxic compounds such as mycotoxins in DDGS might pose a severe threat to animal health. Thus, the content of mycotoxins in DDGS was evaluated in the present study. Aflatoxin B1 (**AFB1**) was not detected, whereas the concentrations of zearalenone (**ZEA**) and deoxynivalenol (**DON**) were 20.6 µg/kg and 400 µg/kg, respectively. The EC of the European Parliament and the Council (May 07, 2002) has established 20 µg/kg as the maximum concentration of AFB1 permissible for all animal feed materials (EC, 2002). Recommendations for the presence of other mycotoxins such as DON and ZEA toxins given in the Commission Recommendation (August 17, 2006) for animal feeding products have established 12,000 µg/kg DON and 3,000 µg/kg ZEA as guidance values for maize by-products (EC, 2006). Therefore, the negative effect of mycotoxins in DDGS was excluded in the current study.

Creswell (2006) reported that 20% DDGS diets lacked sufficient starch, so the birds converted part of the dietary amino acids to glucose to achieve euglycemia and relied increasingly on fatty acid oxidation for energy supply. This is the reason for the reduction in the breast meat yield and the protein content of meat with increasing DDGS levels. Meanwhile, the EE content in DDGS was 11.26% in the current study. The availability of dietary EE and serum TC and TG concentration changed linearly. These changes may influence metabolism over time, and ultimately affect performance. The high proportion of PUFAs in DDGS makes it susceptible to oxidation. Thus, the level of lipid peroxidation (**MDA**) may increase after consuming high levels of corn DDGS (Song and Shurson, 2013). Increasing MDA levels in the liver of birds could affect additional liver functions, including lipid synthesis and transport (Ruan et al., 2017), which may explain the significant increase in the serum TC and TG concentrations after consuming corn DDGS. Supplementation with up to 20% DDGS (Zhai et al., 2020) affected the ability to transport cholesterol from tissues to the liver, as suggested by increased serum TC concentration. Shin et al. (2018) suggested that differences in processing considerably influence the phytochemical content and quality of DDGS. Moreover, due to excessive heat used in drying, thermal abuse can cause lipid oxidation products that may have harmful effects when DDGS incorporated into feeds.

## CONCLUSIONS

In conclusion, the results of the current study provided insight into the precise application of DDGS as an ingredient in Pekin duck diets. Except for consideration of AME, SIDAA, and mycotoxins, further investigation is required on the quality of lipids in DDGS. Inclusion of up to 20% of DDGS in diets increased the diet cost and reduced the growth performance and meat quality of ducks in the present study. Overall, the current study demonstrated that 10% of corn DDGS could be incorporated into the diets of Pekin ducks as an alternative ingredient to corn and soybean meal.
